# Peculiar type 1 congenital pyloric atresia: a case report

**DOI:** 10.1186/1824-7288-36-3

**Published:** 2010-01-14

**Authors:** Enrico Zecca, Mirta Corsello, Claudio Pintus, Lorenzo Nanni, Susanna Zecca

**Affiliations:** 1 Department of Pediatrics, Institute of Pediatrics, Division of Neonatology, Catholic University of the Sacred Heart, Rome, Italy; 2 Department of Pediatrics, Institute of Surgical Pathology, Section of Pediatric Surgery, Catholic University of the Sacred Heart, Rome, Italy; 3 Department of Pediatrics, Tor Vergata University, Rome, Italy

## Abstract

Pyloric atresia (PA) is a very rare condition. Its incidence is approximately 1 in 100,000 newborns and constitutes about 1% of all intestinal atresias. We describe the neonatal course of a peculiar case of type 1 pyloric atresia, in which the pyloric membrane was connected to a second duodenal membrane through a virtual duodenal lumen in a premature newborn. The atypical variant required an unusual side to side gastroduodenostomy. We emphasize the importance of a prompt diagnosis to avoid potentially fatal complications and to warrant a good outcome even in the presence of a strange form of PA in the neonatal period.

## Background

Pyloric atresia (PA) is a very rare condition. Its incidence is approximately 1 in 100,000 newborns and constitutes about 1% of all intestinal atresias [1,2]. Sometimes it occurs with genetic disorders like epidermolysis bullosa and aplasia cutis congenital [3-6] or in association with other atresias of the gastrointestinal tract [7-9]. The presence of associated anomalies is a contributing factor for the reported high mortality [10]. There are 3 recognized anatomic varieties of pyloric atresia: type 1, pyloric membrane (57%); type 2, pyloric canal replaced by solid tissue (34%); type 3, atretic pylorus with a gap between stomach and duodenum (9%) [10]. We describe the neonatal course of a peculiar case of type 1 PA connected to a second duodenal membrane through a virtual lumen in a premature newborn with visualization of polyhydramnios on prenatal ultrasonography. We emphasize the importance of a prompt diagnosis to avoid potentially fatal complications and to warrant a good outcome.

## Case Presentation

### Case report

**Figure 1:**
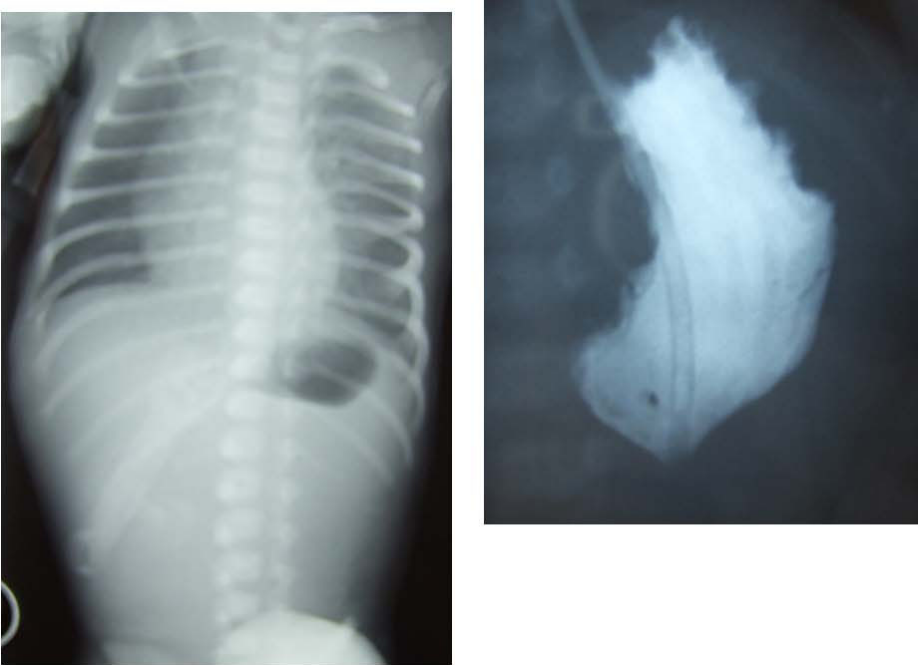
**Pre-operative abdominal X-Ray (left) and contrast study with barium meal (right)**.

A girl was born by emergency cesarean section for abruptio placentae at 34 weeks' gestation and weighted 2130 grams. Apgar scores were 8 and 9 at 1 and 5 minutes, respectively. Pregnancy was complicated by gestational diabetes and polyhydramnios. Physical examination at birth revealed a good-looking, with normal vital data and no signs of sepsis. Routine hematological examinations showed normal values. Nonprojectile and nonbilious vomiting began on the second day of life and persisted for the next day, with staunching > 4 cc/kg after feeding. Feeding was suspended but emesis persisted and the abdomen was mildly distended. A high intestinal obstruction was suspected and an abdominal roentgenogram showed a single gastric bubble with no air in the small and large intestine (Figure 1, **left**). The baby was referred for a surgical opinion on day 3. Contrast study with barium meal confirmed complete obstruction at the pyloric region (Figure 1, **right**). At laparatomy she was found to have a moderately distended stomach and a narrowed duodenal tract. A first membrane was found at the pylorus and a second one was found 2 cm forward, with a virtual duodenal lumen between them. Partial resection of the gastric antrum and of the first duodenal tract was performed, followed by a two layers, side to side gastroduodenostomy.

**Figure 2:**
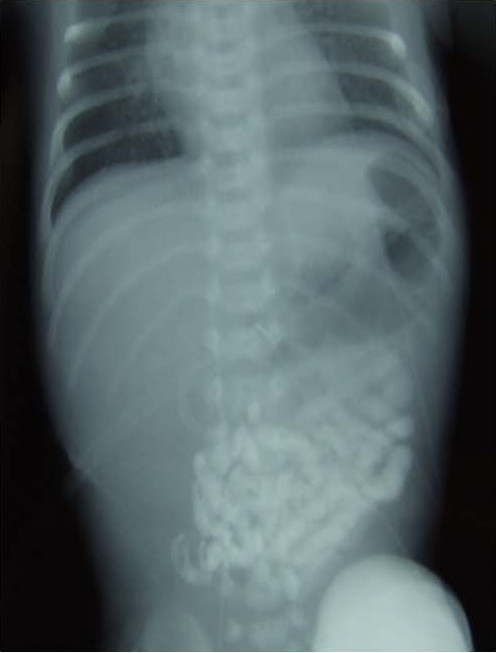
**Postoperative abdominal X-Ray with barium meal**.

The child had an uneventful postoperative course. Ten days postoperatively, a contrast study documented the patency of the entire gastrointestinal tract (Figure 2). She was treated with total intravenous nutrition for 20 days, associated with oral feeding in postoperative day 7 without problems. She was discharged home at 30 days of age on full oral feeding and remains well at 12 months of life.

## Discussion

Pyloric atresia is a very rare condition. Its incidence is approximately 1 in 100.000 newborns and constitutes about 1% of all intestinal atresias [1,2]. Calder reported the first case of PA in 1749 and Touroff performed the first successful operation in 1940 [3,9]. Since then there have been case reports and studies of small series. The etiology of PA is not known but embryologically it is supposed to depend from a developmental arrest between the 5^th ^and 12^th ^week of intrauterine life [8,11]. Familial occurrence with a high frequency of consanguinity and an equal sex incidence suggest a genetic predisposition with an autosomal recessive mode of inheritance [12]. PA can occur as an isolated lesion but in 40-50% of the cases it is associated with other anomalies, epidermolysis bullosa being the commonest. [3-6] PA may be also part of hereditary multiple intestinal atresias (HMIAs) involving the stomach, duodenum, jejunum, ileum, colon, and rectum. This form has an extremely rare occurrence and a pathogenesis that is still speculative, including the possibility of a combined immunodeficiency syndrome [7-9]. The presence of associated anomalies is a contributing factor for the reported high mortality [10]. Our case can be considered a variant of type 1 because of the presence of a second membrane located in the first tract of duodenum and of a virtual duodenal lumen between the two membranes.

Antenatal diagnosis may be difficult and in our case only suspected by the presence of polyhydramnios, which is associated with PA in more than 50% of cases, with a dilated stomach but in the absence of a double bubble [13]. The clinical features of PA are well documented but the diagnosis may be delayed because the neonates usually are well at the time of first presentation. Typically, they develop nonbilious vomiting and abdominal distension during the first days of life and examination may show gastric peristalsis. A delayed diagnosis may lead to pulmonary aspiration, severe metabolic derangement and gastric perforation, which can be fatal. The instrumental diagnosis of PA is made on plain abdominal x-ray, based on the presence of a single large gastric air bubble with no gas distally. This was confirmed in our patient by a barium meal.

Different operative procedures can be used, depending on the anatomic type. In the literature, the best results from operative treatment of typical type 1 and type 2 PA were obtained by excision of the membrane and pyloroplasty according to Heineke-Mikulicz or Finney [2,14]. Pyloro-duodenostomy is the treatment of choice in case of type 3 PA. In our patient, the association of a duodenal membrane connected with the pyloric one by a virtual lumen required an unusual side to side gastroduodenostomy. The prognosis of PA is variable. The overall mortality is very high exceeding 50% but it is due to the high incidence of severe and often fatal associated anomalies [6]. Isolated PA and PA associated with other intestinal atresias can be managed successfully. Early diagnosis and surgery, together with current neonatal supportive care, have significantly improved the survival rate in these patients.

## Conclusion

Our case demonstrates that a prompt diagnosis is crucial to obtain a good outcome, even in the presence of a strange form of PA in the neonatal period.

## Consent

Written informed consent was obtained from the patient for publication of this case report and any accompanying images. A copy of the written consent is available for review by the Editor-in-Chief of this journal."

## Competing interests

The authors declare that they have no competing interests.

## Authors' contributions

**EZ **took care of the general presentation and the final version of the manuscript

**MC **got all the information about the case and wrote the first draft of the manuscript

**CP **and **LN **were the surgeons involved, they gave the pictures and all details concerning the surgical procedures

**SZ **made an extensive literature review and revised the English version.

We declare that all authors read and approved the final manuscript
